# Creating and Validating a DNA Methylation-Based Proxy for Interleukin-6

**DOI:** 10.1093/gerona/glab046

**Published:** 2021-02-17

**Authors:** Anna J Stevenson, Danni A Gadd, Robert F Hillary, Daniel L McCartney, Archie Campbell, Rosie M Walker, Kathryn L Evans, Sarah E Harris, Tara L Spires-Jones, Allan F McRae, Peter M Visscher, Andrew M McIntosh, Ian J Deary, Riccardo E Marioni

**Affiliations:** 1 Centre for Genomic and Experimental Medicine, Institute of Genetics and Molecular Medicine, University of Edinburgh, UK; 2 UK Dementia Research Institute, Edinburgh Medical School, University of Edinburgh, UK; 3 Centre for Clinical Brain Sciences, Edinburgh BioQuarter, UK; 4 Lothian Birth Cohorts, University of Edinburgh, UK; 5 Department of Psychology, University of Edinburgh, UK; 6 Centre for Discovery Brain Sciences, University of Edinburgh, UK; 7 Institute for Molecular Bioscience, The University of Queensland, Brisbane, Australia; 8 Division of Psychiatry, University of Edinburgh, Royal Edinburgh Hospital, UK

**Keywords:** Cognitive ability, DNA methylation, Epigenetics, Inflammation, Interleukin-6

## Abstract

**Background:**

Studies evaluating the relationship between chronic inflammation and cognitive functioning have produced heterogeneous results. A potential reason for this is the variability of inflammatory mediators which could lead to misclassifications of individuals’ persisting levels of inflammation. DNA methylation (DNAm) has shown utility in indexing environmental exposures and could be leveraged to provide proxy signatures of chronic inflammation.

**Method:**

We conducted an elastic net regression of interleukin-6 (IL-6) in a cohort of 875 older adults (Lothian Birth Cohort 1936; mean age: 70 years) to develop a DNAm-based predictor. The predictor was tested in an independent cohort (Generation Scotland; *N* = 7028 [417 with measured IL-6], mean age: 51 years).

**Results:**

A weighted score from 35 CpG sites optimally predicted IL-6 in the independent test set (Generation Scotland; *R*^2^ = 4.4%, *p* = 2.1 × 10^−5^). In the independent test cohort, both measured IL-6 and the DNAm proxy increased with age (serum IL-6: *n* = 417, β = 0.02, *SE* = 0.004, *p* = 1.3 × 10^−7^; DNAm IL-6 score: *N* = 7028, β = 0.02, *SE* = 0.0009, *p* < 2 × 10^−16^). Serum IL-6 did not associate with cognitive ability (*n* = 417, β = −0.06, *SE* = 0.05, *p* = .19); however, an inverse association was identified between the DNAm score and cognitive functioning (*N* = 7028, β = −0.16, *SE* = 0.02, *p*_FDR_ < 2 × 10^−16^).

**Conclusions:**

These results suggest methylation-based predictors can be used as proxies for inflammatory markers, potentially allowing for further insight into the relationship between inflammation and pertinent health outcomes.

Acute inflammation is a necessary component of the biological response to harmful stimuli. However, there is increasing recognition that a chronic, subacute elevation of serum concentrations of proinflammatory mediators occurring in older age may underpin the development of many age-related diseases, including cancer, atherosclerosis, and Alzheimer’s disease (1–4). In spite of this, studies of chronic inflammation and its therapeutics, particularly in cognitive decline and dementia, have produced heterogeneous results (5,6).

Population studies investigating the relationship between chronic inflammation and incident morbidities typically rely on a single measurement of circulating inflammatory biomarker levels as proxies for individuals’ persistent inflammatory states. Interleukin-6 (IL-6), a pleiotropic proinflammatory cytokine, is a principal stimulator of an extensive range of acute-phase inflammatory proteins, including C-reactive protein (CRP), serum amyloid A, and fibrinogen (7). It has been hypothesized that IL-6 is critical in the transition from the acute, beneficial inflammatory response, to chronic and deleterious inflammation, marking it as a key target for research into the process (8). However, inflammatory cytokines are vulnerable to analytical measurement error; specifically, circulating plasma IL-6 levels can show transient variability due to manifold influences, including the obvious precipitate—infection—but also diet and activity levels (9,10). Because of this, the concentration of IL-6 within the blood can rapidly fluctuate meaning a single measurement of its serum levels may not accurately reflect an individual’s basal or enduring levels of the cytokine. This is an important consideration when utilizing it as a surrogate of chronic, rather than acute, inflammation (11,12).

Epigenetic mechanisms afford an opportunity to further understand the molecular pathophysiology of inflammation. DNA methylation (DNAm), the most commonly studied epigenetic mechanism, is involved in the regulation of gene expression and chromosomal stability (13). It has been proposed as a putative intermediary, linking inflammation with sequent disease outcomes, with differential DNAm profiles identified in inflammatory diseases (14–16). Studies are beginning to focus on resolving the link between inflammation and DNAm (17–19); however, there remains a relative dearth of understanding about the association between DNAm and proinflammatory cytokines, and of how this information could be used to inform disease progression or risk stratification. Recently, DNAm alterations have been leveraged to provide peripheral predictors, or biomarkers, of disease status and as archives of habitual traits (20,21). These indices have demonstrated potential utility in epidemiological research contexts (22).

DNAm is considered relatively stable in the short term, and thus using it to proxy IL-6 may allow for a more reliable indicator of enduring levels of inflammation, and indeed provide a proxy measurement of the biomarker in studies in which levels of the cytokine are not available. In this study, we utilize DNAm data from the Lothian Birth Cohort 1936 (LBC1936) to train an optimized predictor of IL-6 using a penalized regression model. This DNAm IL-6 score was subsequently applied to a large out-of-sample cohort (Generation Scotland [GS]) to (i) characterize its relationship with measured IL-6 and IL-6 correlates, and (ii) determine its association with cognitive ability, a trait correlated with a variety of health outcomes.

## Method

### The Lothian Birth Cohort 1936

The LBC1936 is a longitudinal study of ageing comprising individuals born in 1936. Most of them took part in the Scottish Mental Survey 1947 in which a test of cognitive ability was administered to children attending school in Scotland at age around 11 years. Participants living in Edinburgh and the Lothians were re-contacted around 60 years later, with 1091 individuals consenting to join the LBC1936 study. At recruitment, participants were aged around 70 years and subsequently have completed up to 4 waves of detailed testing, repeated triennially. Comprehensive genetic, epigenetic, cognitive, psychosocial, health and lifestyle, and biomarker data are available for these individuals across the eighth decade. Full details of the recruitment and testing protocol for the study have been described previously (23,24). Ethical permission for the LBC1936 was obtained from the Multi-Centre Research Ethics Committee for Scotland (MREC/01/0/56) and the Lothian Research Ethics Committee (LREC/2003/2/29). Written informed consent was obtained from all participants.

#### LBC1936 DNAm preparation

DNA extracted from whole blood was analyzed using the Illumina 450K BeadChip methylation array at the Edinburgh Clinical Research Facility. Details of quality control procedures have been described previously (25,26). Briefly, raw intensity data were background-corrected and normalized using internal controls. Low-quality samples (those exhibiting inadequate bisulfite conversion, hybridization, or nucleotide extension) were removed upon manual inspection. Quality control further removed probes with a low detection rate (*p* > .01) and samples with a low call rate (<450 000 probes detected at *p*-value <.01). Finally, samples with a mismatch between genotype and SNP control probes or DNAm-predicted and reported sex, were additionally removed. Methylation data was subset to probes in common on both the 450k and EPIC arrays (*n* = 428 489). DNAm data were available for 889 individuals at Wave 1 of the study (aged ~70 years).

#### Phenotype preparation

Blood plasma samples were collected from LBC1936 participants at Wave 1 of the study and were analyzed using a 92-plex proximity extension assay (Olink Bioscience, Uppsala, Sweden). This represents the Olink inflammation panel which provides a multiplex immunoassay, facilitating the analysis of 92 inflammation-related protein biomarkers. We focused analyses on IL-6 as this was measured in the prediction cohort (see “Out-of-Sample Prediction Cohort: GS” section) and is widely used in studies of inflammation and cognitive function. Briefly, the Olink protocol involves the incubation of 1 μL of plasma sample with proximity antibody pairs linked to DNA reporter molecules. Once a complementary antibody has bound to the antibody pair, the DNA tails form an amplicon by proximity extension which is subsequently quantified using high-throughput real-time polymerase chain reaction. Data underwent pre-processing by Olink using NPX Manager software. Prior to analyses, IL-6 levels were rank-based inverse normalized to correct for skewness, and normalized protein levels were then regressed onto age, sex, 4 genetic principal components, and Olink array plate. Standardized residuals from this regression model were used in the penalized regression analysis. Complete DNAm and IL-6 data were available for 875 individuals.

#### Development of a DNAm-based predictor of IL-6 in LBC1936

An elastic net penalized regression was run to derive the DNAm-based predictor of IL-6. This method is commonly applied to DNAm data and helps to control for collinearity in predictor variables. It is a particularly useful method when dealing with a large number of predictors. The elastic net penalty is an intermediate of LASSO and ridge regression—the LASSO approach yields a sparse solution with a minimal set of non-zero coefficients from the feature set (it will include one feature from a set of correlated features) and ridge regression shrinks the coefficients for correlated features toward each other (so includes a large number of features).

The elastic net model was run using the *glmnet* library in R (27), with 12-fold cross-validation. To reduce overfitting, folds were specified by methylation analysis batch, yielding between 63 and 83 observations per fold. IL-6 and DNAm levels at 428 489 CpG sites were entered as the dependent and independent variables, respectively. In order to apply the relevant penalty, the mixing parameter (α) was set to .5. Coefficients for the model with the lambda value (regularization parameter) corresponding to the minimum mean cross-validated error were extracted. These weights were then applied to the corresponding CpGs in an out-of-sample prediction cohort in order to derive the DNAm-based predictor of IL-6.

### Out-of-Sample Prediction Cohort: GS

The GS cohort was used for external predictions. GS is a family-structured, population-based genetic epidemiology cohort comprising ~22 000 participants aged between 18 and 99 years. These individuals have been widely profiled in regards to cognitive ability, health, genetics, and epigenetics, further details of which can be found elsewhere (28). All components of GS received ethical approval from the NHS Tayside Committee on Medical Research Ethics (REC reference number: 05/S1401/89). GS has also been granted Research Tissue Bank status by the Tayside Committee on Medical Research Ethics (REC reference number: 10/S1402/20), providing generic ethical approval for a wide range of uses within medical research.

#### DNAm in GS

The DNAm arrays in GS were run in 2 separate sets. The present study includes analysis of methylation data from 7028 unrelated individuals in total (2578 unrelated individuals in the first set and 4450 in the second set—unrelated to both each other and the first set). Methylation was quantified from whole blood using the Illumina HumanMethylationEPIC BeadChip. Quality control steps for set 1 have been reported previously (29). Briefly, samples were removed (i) if they were deemed as outliers based on a visual inspection of the log median intensity of the methylated versus unmethylated signal per array, (ii) if there was a mismatch between their predicted and reported sex, and/or (iii) if ≥1% of CpGs had a detection *p*-value >.05. Probes were removed based on (ii) a bead count <3 in >5% of samples, and (ii) ≥5% of samples having a detection *p*-value >.05. CpGs with missing values, non-CpG probes, and nonautosomal probes were additionally removed. Quality control steps for set 2 have also been reported previously and were near identical to the set 1 protocol (30). Proportions of granulocytes, natural killer cells, B cells, CD4^+^ T cells, and CD8^+^ T cells were estimated using the Houseman method in *minfi* (31,32).

#### Phenotype preparation in GS

##### Serum IL-6.

—Serum IL-6 was quantified at the University of Glasgow using a high-sensitivity commercial enzyme-linked immunosorbent assay (R&D Systems, Oxon, UK). IL-6 data were available for 417 individuals. These samples had previously been selected as father/offspring pairs in a telomere length study. The data in the current study were subset to exclude any related individuals. This limited the number of samples from older females in the data set. To correct for positive skew, IL-6 data were log-transformed (natural log) prior to analyses.

##### Correlates of IL-6.

—Several modifiable and nonmodifiable factors have been shown to associate with circulating IL-6 levels, including smoking (33), alcohol intake (34), body mass index (BMI; (35)), and socioeconomic status (36). These were assessed in GS as follows: smoking was divided into current smokers, ex-smokers (split into those who had quit within the previous 12 months of their blood sample date, and those who had quit prior to that), and never smokers; alcohol consumption (units) in a typical week; BMI was calculated as the ratio of weight in kilograms divided by height in meters squared; and social deprivation was measured using the Scottish Index of Multiple Deprivation (SIMD—www.simd.scot). The SIMD ranks geographical areas in Scotland based on current income, employment, health, education, skills and training, geographic access to services, housing, and crime. The SIMD provides a standardized measure of relative deprivation throughout Scotland. To reduce positive skew, a log(units+1) transformation was used for the alcohol consumption data prior to analysis.

Due to the inclusion of CpG sites that have previously been related to smoking in the DNAm IL-6 score, we additionally generated a methylation-based smoking score using the *EpiSmokEr* R package (37). *EpiSmokEr* provides a smoking score derived from methylation levels at 187 CpG sites based on an EWAS of current versus never smokers (38,39).

##### Cognitive tests.

—To derive a general cognitive ability phenotype, a principal component analysis was applied to the cognitive tests of executive function (verbal fluency), processing speed (digit-symbol coding), and logical memory. Scores on the first un-rotated principal component were used as a general fluid-type cognitive ability score for each individual in GS_._ This component accounted for 50% of the variance and individual test loadings ranged from 0.63 to 0.77. Full details on the cognitive assessments for the GS cohort have been described previously (40,41).

### Statistical Analysis

Pearson correlations were calculated between serum IL-6 and the DNAm IL-6 score and between the latter and the imputed immune cell proportions (CD8^+^ T cells, CD4^+^ T cells, natural killer cells, B cells, monocytes, and granulocytes). The relationship between serum and DNAm IL-6 and the estimated immune cell proportions was further investigated in linear regression models. Here, the difference between the *R*^2^ statistic from a basic model (IL-6 ~ age + sex), and the same model adjusted for the imputed immune cell proportions, was calculated to estimate the proportion of variance accounted for by these predictors.

To investigate the validity of the DNAm IL-6 score, linear regression models were run with phenotypes that have previously been associated with circulating IL-6 levels: BMI, alcohol intake, and social deprivation. Ordinal logistic regression models were run for smoking. Measured IL-6 models were adjusted for age and sex, with the DNAm IL-6 models further adjusted for methylation set and the cell proportions imputed from the methylation data. In a sensitivity analysis, each of the models was re-run adjusting for a methylation-predicted smoking score using the *EpiSmokEr* output.

Linear regression models were used to establish the association of both measured IL-6 and the DNAm IL-6 score with age and with cognitive ability. Age (as a predictor of interest or a covariate) and sex were included in both models. The DNAm IL-6 model was further adjusted for methylation set and estimated cell proportions. The cognitive ability model was repeated after adjustment for the methylation smoking score. All analyses were conducted in the R statistical environment version 3.5.0 (42). Correction for multiple testing was applied using the false discovery rate (FDR *p* < .05) across all tests.

## Results

### Cohort Information

Summary information for all variables included in analyses is presented in Table 1. LBC1936 is an older cohort than GS (LBC1936 Wave 1 mean age: 69.6 years; GS mean age: 50.8 years) with a more even balance of the sexes (LBC1936: 49.4% female; GS: 58.2% female). LBC1936 had a higher mean IL-6 level compared to GS (LBC1936 mean: 3.99; GS mean: 1.8).

**Table 1.** Summary of the Lothian Birth Cohort 1936 (Wave 1) and Generation Scotland Variables

	LBC1936			GS		
	*n*	Mean	*SD*	*n*	Mean	*SD*
Age (y)	895	69.6	0.8	7028	50.8	12.9
Females (%)	437 (49.9)	—	—	4089 (58.2)	—	—
IL-6 (pg/mL)	875	3.99	1.87	417	1.8	1.9
General cognitive ability	—	—	—	6876	0.007	0.99
Logical memory				6953	30.8	7.9
Digit-symbol coding	—	—	—	6952	70.7	16.6
Verbal fluency	—	—	—	6949	40.6	11.7
Alcohol (average units/wk)	—	—	—	6414	10.5	11.02
BMI (kg/m^2^)	—	—	—	6988	26.9	5.09
Smoking status (%)	—	—	—	6854	—	—
Current				1135 (16%)	—	—
Ex				2167 (32%)	—	—
Never				3552 (52%)	—	—
SIMD	—	—	—	6673	3953	1858

*Note*: BMI = body mass index; GS = Generation Scotland; IL = interleukin; LBC1936 = Lothian Birth Cohort 1936; SIMD = Scottish Index of Multiple Deprivation.

### Elastic Net Regression in LBC1936

The elastic net model returned 35 CpG sites. The probes and their relevant weights are presented in Supplementary Table 1. A DNAm proxy for IL-6 was generated in GS by multiplying methylation beta values with the coefficients from this output, and summing into a single score.

The CpG sites selected by the model spanned genes with a range of functions, including oxidative DNA damage repair and glucose and insulin homeostasis. Methylation at one of the probes included in the score (cg05575921, *AHRR*) has been strongly (inversely) associated with smoking status (43–46), with 7 further probes (cg04583842, cg04381957, cg12503394, cg14044707, cg25250132, cg17412005, and cg20059928), showing similar, though weaker, associations (Supplementary Table1) (47,48). Additionally, a number of the CpG sites included in the predictor have previously been associated with gestational age and carcinoma, both of which have also been associated with IL-6 levels and regulation (49,50).

### Correlation With IL-6 in GS

**Figure 1.**
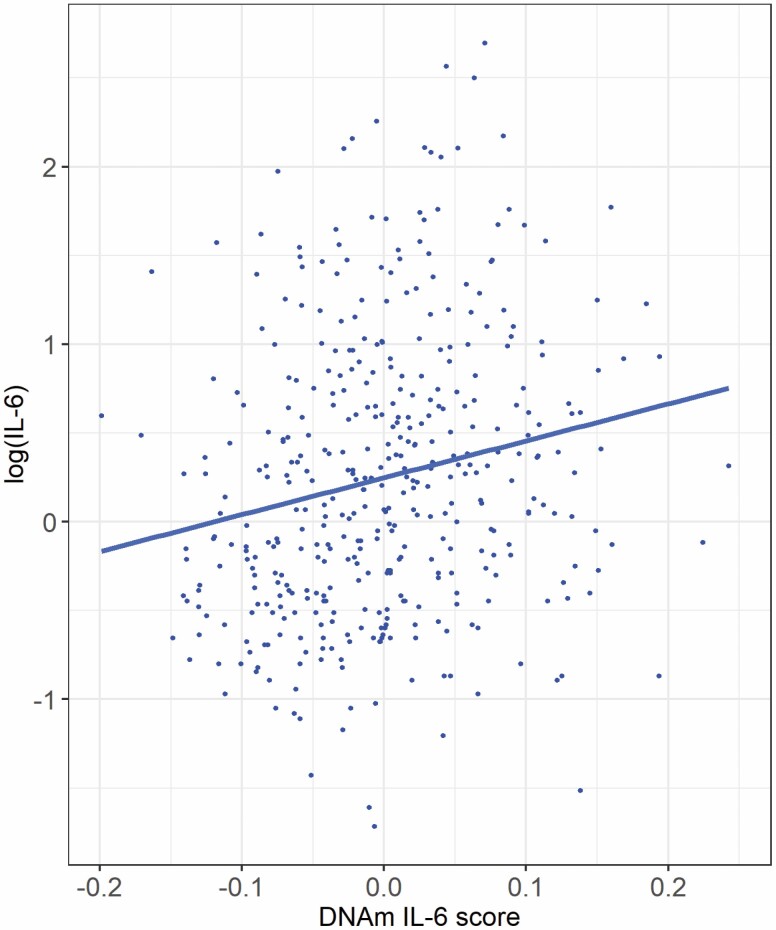
Plot of the relationship between log(IL-6) and the DNAm IL-6 score in the subset of the Generation Scotland data set in which serum IL-6 was measured (*n* = 417).

To test the predictive performance, Pearson correlations were calculated between the DNAm IL-6 score and measured IL-6 in GS. The correlation coefficient between measured log(IL-6) and the elastic net-derived IL-6 score was .21 (*p* = 2.1 × 10^−5^, *R*^2^ = 4.4%). A plot of serum IL-6 versus this DNAm IL-6 score is presented in Figure 1. Four of the CpG sites comprising the score (cg17412005, cg21368161, cg04583842, and cg04381957) showed a similar, or slightly stronger, correlation with serum IL-6 compared to the composite DNAm IL-6 score (Supplementary Table 2). In LBC1936, the 35 CpGs correlated between 0.12 and 0.18 with serum IL-6, with the strongest correlations observed for cg17412005 and cg12929678 (Supplementary Table 1).

Because of the association between some of the CpG sites included in the DNAm IL-6 score and smoking, the association between the DNAm IL-6 score and log(IL-6) was re-run in a regression analysis, adjusting for a methylation-based score for smoking. In the unadjusted model, the DNAm IL-6 score was significantly positively associated with log(IL-6) (β = 0.16, *SE* = 0.04, *p* = 2.1 × 10^−5^). Adjusting for the methylation smoking score had little effect on this relationship (β = 0.15, *SE* = 0.04, *p* = 1.6 × 10^−4^), suggesting the association between measured IL-6 and the DNAm IL-6 score is largely not influenced by smoking. A correlation plot of log(IL-6), the DNAm IL-6 score, and the methylation-predicted smoking score is presented in Supplementary Figure 1.

### Correlation With Cell Proportions in LBC1936 and GS

The correlation between the DNAm IL-6 score and the estimated leukocyte proportions is presented in Supplementary Figure 2. These ranged from −0.68 (CD4^+^ T cells) to 0.38 (granulocytes). Due to the high correlations identified here, we additionally tested the association between the imputed cell proportions with measured IL-6 in both LBC1936 (*N* = 875) and GS (*n* = 417). In LBC1936, the incremental *R*^2^ statistic (representing the difference between a model adjusted for age and sex and a model adjusted for age, sex, and the 6 cell proportions) was .042 and in GS, it was .087. In GS, the incremental *R*^2^ for the DNAm IL-6 score was .48. This suggests that a greater proportion of variance was explained by the cell proportions in the DNAm IL-6 score compared to serum IL-6.

### Association With Age in GS

**Figure 2.**
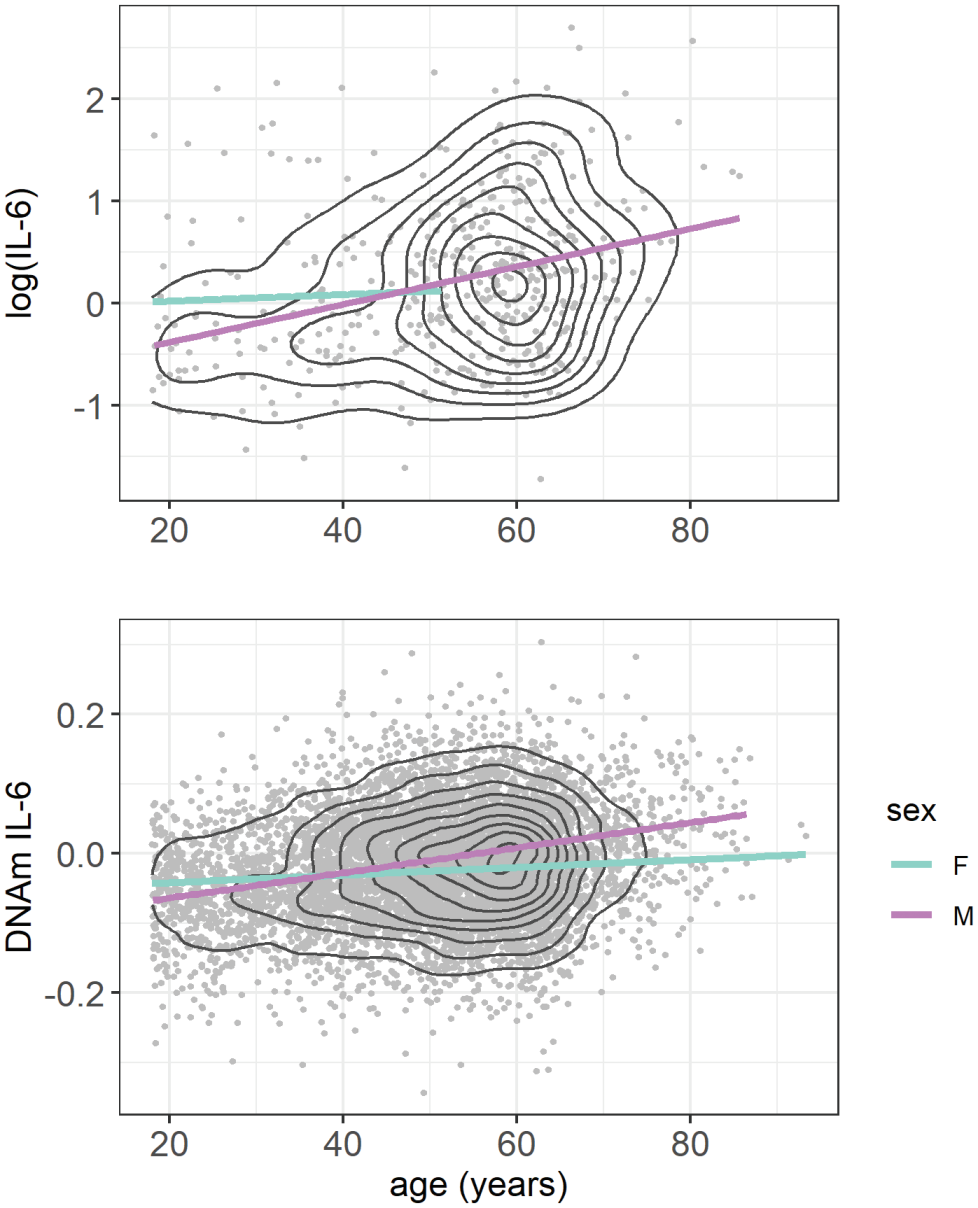
Plots of log(IL-6) (*n* = 417) and DNAm IL-6 score (*N* = 7028) with chronological age in Generation Scotland. The measured IL-6 samples had previously been selected as father/offspring pairs limiting the number of samples from older females in the dataset. Gray dots represent individual participant measurements with regression lines shown for females and males in purple and blue, respectively. Black lines represent density.

As chronic inflammation is a hallmark of older age, we examined the association between the measured IL-6 phenotype and the DNAm IL-6 score with chronological age. Plots of these associations are presented in Figure 2. Both serum IL-6 and the DNAm IL-6 scores were found to increase as a function of age (serum IL-6: β = 0.022, *SE* = 0.004, *p* = 1.3 × 10^−7^; DNAm IL-6 score: β = 0.015, *SE* = 0.0009, *p* < 2 × 10^−16^). No significant effect of sex was identified in the serum IL-6 dynamics; however, males were found to have a higher DNAm IL-6 score compared to females (β = 0.25, *SE* = 0.02, *p* < 2 × 10^−16^). Furthermore, inclusion of an interaction term between age and sex indicated the DNAm IL-6 score increased more rapidly over time in males compared to females (β = 0.016, *SE* = 0.002, *p* < 2 × 10^−16^).

### Association With IL-6 Correlates in GS

The associations between both serum log(IL-6) and the DNAm IL-6 score with factors previously associated with IL-6 levels are presented in Table 2. For comparison, the associations between serum log(IL-6) and the same factors in LBC1936 are presented in Supplementary Table 3.

**Table 2.** Associations Between Traits Previously Correlated With Circulating IL-6, With Both Measured log(IL-6) and the DNAm IL-6 Score in Generation Scotland

	Log(IL-6) (*n* = 417)				DNAm IL-6 Score—Subset With Measured IL-6 (*n* = 417)				DNAm IL-6 Score—Full Data set (*N* = 7028)			
	β	*SE*	*p*	*p*_FDR_	β	*SE*	*p*	*p*_FDR_	β	*SE*	*p*	*p*_FDR_
BMI	0.21	0.04	**1.2 × 10** ^**−6**^	**3.2 × 10** ^**−6**^	0.15	0.08	.073	.12	0.088	0.02	**8.3 × 10** ^**−7**^	**3.0 × 10** ^**−6**^
SIMD	−0.051	0.05	.28	.38	0.04	0.08	.60	.62	−0.24	0.02	**<2 × 10** ^**−16**^	**<2 × 10** ^**−16**^
Alcohol	0.035	0.05	.51	.59	0.11	0.08	.20	.27	0.079	0.02	**1.0 × 10** ^**−5**^	**2.6 × 10** ^**−5**^
Smoking	0.15	0.09	.11	.19	0.82	0.15	**5.5 × 10** ^**−8**^	**2.4 × 10** ^**−7**^	1.20	0.04	**<2 × 10** ^**−16**^	**<2 × 10** ^**−16**^

*Note*: BMI = body mass index; SIMD = Scottish Index of Multiple Deprivation. Log odds are presented for smoking. Significant associations are highlighted in bold.

In the full GS cohort (*N* = 7028), the DNAm IL-6 score was positively associated with BMI (β = 0.09, *SE* = 0.02, *p*_FDR_ = 3.0 × 10^−6^), self-reported smoking status (log odds = 1.2, *SE* = 0.04, *p*_FDR_ < 2 × 10^−16^), and alcohol intake (β = 0.08, *SE* = 0.02, *p* = 2.6 × 10^−5^), and negatively with social deprivation, such that a higher DNAm IL-6 score associated with greater deprivation (β = −0.24, *SE* = 0.02, *p*_FDR_ < 2 × 10^−16^).

In the subset of the cohort with measured IL-6 (*n* = 417), the cytokine was positively associated with BMI (β = 0.21, *SE* = 0.04, *p* = 3.2 × 10^−6^), but not with self-reported smoking status, social deprivation, or alcohol intake (*p* ≥ .19). The direction of association was consistent with findings with serum IL-6 in LBC1936, with the exception of alcohol consumption, although *p* >.05 across all cohorts for this variable (Supplementary Table 3). In this GS subset, the association between the DNAm IL-6 score and smoking status remained (log odds: 0.82, *SE* = 0.15, *p*_FDR_ = 2.4 × 10^−7^), but the associations with BMI, alcohol intake, and social deprivation were no longer significant (*p* ≥ .07).

Given the composition of the DNAm IL-6 score, the associations with smoking status were unsurprising. As a sensitivity analysis, we re-ran the models assessing the relationship with BMI, alcohol intake, and social deprivation adjusting for methylation-predicted smoking to test for potential confounding. Here, the association with BMI was strengthened (β = 0.16, *SE* = 0.02, *p*_FDR_ = 1.3 × 10^−13^, increase = 78%), whereas the association with social deprivation was attenuated but remained significant (β = −0.07, *SE* = 0.02, *p*_FDR_ = 8.7 × 10^−4^, attenuation = 71%). The association with alcohol intake was no longer significant (*p* = .5).

### Association With Cognitive Ability in GS

An inverse association between cognitive ability and the DNAm IL-6 score was identified in the full GS cohort (*N* = 7028, β = −0.16, *SE* = 0.02, *p*_FDR_ < 2 × 10^−16^). Following adjustment for methylation-based smoking (in addition to age, sex, methylation set, and the imputed immune cells), the association was attenuated but remained significant (β = −0.08, *SE* = 0.02, *p*_FDR_ = 5.1 × 10^−7^, attenuation = 50%).

In the subset of the cohort with serum IL-6 data (*n* = 417), IL-6 itself was not significantly associated with cognitive ability (β = −0.06, *SE* = 0.05, *p* = .19), but the DNAm IL-6 score showed a negative association with cognitive function (β = −0.19, *SE* = 0.07, *p*_FDR_ = .014).

## Discussion

In the current study, we created a poly-epigenetic score of the proinflammatory cytokine IL-6 based on results from an elastic net penalized regression. This DNAm IL-6 score was found to increase with age, and associate with IL-6 correlates and general fluid-type cognitive ability.

The DNAm IL-6 score highlights the potential utility of exploiting methylation data to predict biological outcomes. The magnitude of the association between the DNAm IL-6 score and serum IL-6 was relatively modest. However, given its temporal variability, IL-6 itself may be an unreliable signature of chronic inflammation. If the composite epigenetic score better tracks this chronicity, a moderate association between the two may be expected. Integration of information from multiple CpG sites may provide a more reliable estimate of chronic inflammation, overcoming the periodic instability of the cytokine itself. However, four of the CpG sites alone displayed a similar relationship to IL-6 as the DNAm score. Future studies should replicate this analysis to determine if a single CpG site could be as predictive as a composite score. We identified strong correlations between the estimated leukocyte proportions and the DNAm IL-6 score, suggesting a relatively large proportion of the variance in the latter is explained by the former. This is perhaps unsurprising given the interplay between inflammation and the immune system. IL-6 is synthesized by multiple cell types including neutrophils, B cells, and macrophages in response to infectious or immunologic stimuli. Further, IL-6 enacts a pleiotropic effect on the immune response, including inducing differentiation of B cells into antibody-producing cells (51), CD4^+^ T cells into subsets of effector T helper cells (52), and CD8^+^ T cells into cytotoxic T cells (53). However, the incremental R2 of the models assessing the association with measured IL-6 were small in comparison. This indicates a contrast between serum IL-6 and the DNAm IL-6 score, with the methylation score more directly tracking alterations in cell proportions. Determining the causal pathway of this association is challenging, given that both the DNAm IL-6 score and cell proportions were predicted from the methylation data. Assessing the relationship between the DNAm IL-6 score and measured cell counts would be beneficial to establish if the association identified here was replicated.

IL-6 is a widely used marker of inflammation and circulating levels of the cytokine typically rise in older age (54,55). From these cross-sectional data, we identified higher DNAm IL-6 scores with age, with males exhibiting an accelerated pseudo-trajectory compared to females. Though this sex effect was not identified in the serum IL-6 pseudo-trajectories, it has been reported previously (56), and may have been missed here due to the lack of IL-6 data available from older females in the GS cohort. The DNAm IL-6 score showed associations with BMI, social deprivation, alcohol intake, and smoking status—all established correlates of IL-6. The robust association identified with smoking status was unsurprising, given the inclusion of the smoking-associated probes in the DNAm IL-6 score. In the sensitivity analysis, the association with social deprivation was partly attenuated when adjusting for the methylation-based smoking score; however, it remained significant. No confounding was identified in the association with BMI.

Serum concentrations of IL-6 have repeatedly been linked to age-related morbidities, including cancer and frailty (55). Though IL-6, and inflammation in general, has additionally previously been associated with cognitive ability and decline in older adults, there remains much heterogeneity in the literature assessing the relationship. A potential reason for this is the aforementioned acute nature of inflammatory mediators and differences in assays used to quantify them. We found that the DNAm IL-6 score associated with general cognitive ability in the full GS cohort (*N* = 7028). This echoes previous findings in the same cohort with a similarly constructed DNAm score for CRP, in which no association was identified between serum CRP and cognitive ability, but an inverse association was found between the latter and the DNAm CRP score (57). This further demonstrates the potential value and applicability of proxy methylation-based scores for phenotypes that are either not available, or may in themselves be relatively unreliable. Though CRP and IL-6 are the most commonly utilized inflammatory markers in studies of the relationship between inflammation and cognitive ability, inflammation represents a complex cascade, involving a multitude of immune cells, proteins, and molecular mediators, each potentially associated with, or impacting, methylation and cognitive function. Creating DNAm scores for a panel of inflammatory biomarkers and examining their association with cognitive performance would be beneficial to establish their relative importance. It seems the association between the DNAm IL-6 score and cognitive ability was, at least in part, driven by smoking, which has previously been associated with poorer cognitive health (58). However, though attenuated, the association did remain significant upon adjustment for the methylation-based smoking score, suggesting some independent association with the DNAm IL-6 score. Further investigation of these associations in older cohorts and those with longitudinal measures available would help to clarify the relationship, and additionally establish if the DNAm IL-6 score associates with cognitive decline.

This is the first study to develop and characterize a DNAm-based predictor of the proinflammatory cytokine IL-6. The utility of this proxy phenotype is particularly evident for studies in which DNAm is quantified but for which IL-6 is unavailable. Additionally, utilizing a composite methylation score that integrates information from multiple sites could potentially provide a more reliable estimate of chronic inflammation, allowing for clearer insight when assessing its relation to health outcomes. This study is limited by the relatively small number of serum IL-6 samples available in the GS cohort, particularly from older females. Further, the DNAm IL-6 score was developed in a birth cohort with a narrow age range, all of whom have survived into the eighth decade. This may have limited the ability of the score to predict into a cohort with a broader age range and, potentially, more variable health. This is an important consideration when utilizing the score in future analyses in different cohorts. To further validate the DNAm IL-6 score, assessing its relationship to serum IL-6 in large cohorts with repeat measures from the same individual would be valuable. Finally, whereas we examined the association between the DNAm IL-6 score and previous correlates of IL-6, we acknowledge that these variables are likely to additionally correlate with DNAm, making the associations challenging to delineate.

In summary, we have illustrated that utilizing DNAm data can provide a proxy for the proinflammatory cytokine IL-6 that associates with pertinent health, lifestyle, and cognitive outcomes. Future studies with DNAm data, inflammatory disease measures, and longitudinal follow-up available will allow for further insight into the utility of this DNAm-based predictor and its ability to act as a measure of chronic inflammation compared to the labile phenotype.

## Data Availability

LBC1936 data are available on request from the Lothian Birth Cohort Study, University of Edinburgh (Simon Cox, simon.cox@ed.ac.uk). LBC data are not publicly available due to them containing information that could compromise participant consent and confidentiality. According to the terms of consent for Generation Scotland participants, access to data must be reviewed by the Generation Scotland Access Committee. Applications should be made to access@generationscotland.org. Analysis code is available at: gitlab.com/marioni-group/il-6_elasticnet.

## Supplementary Material

glab046_suppl_Supplementary_eFigure1

glab046_suppl_Supplementary_eFigure2

glab046_suppl_Supplementary_Material
