# Supplementary figures and images for: Creating and Validating a DNA Methylation-Based Proxy for Interleukin-6
Source: J Gerontol A Biol Sci Med Sci. 2021 Feb 17;76(12):2284–92. doi: 10.1093/gerona/glab046 (PMC8599002; doi:10.1093/gerona/glab046)

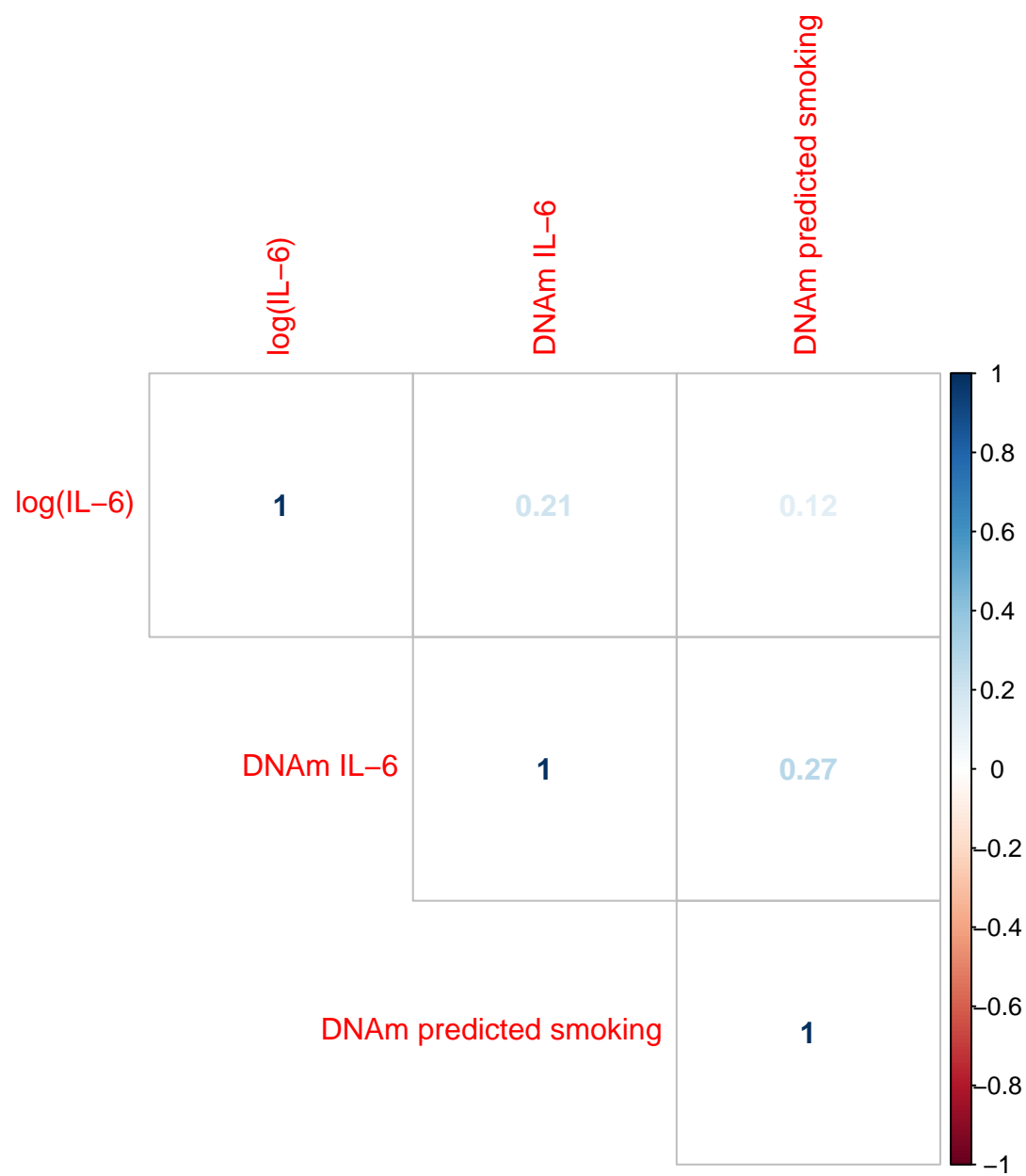

Supplement: glab046_suppl_Supplementary_eFigure1 [file glab046_suppl_supplementary_efigure1.pdf]

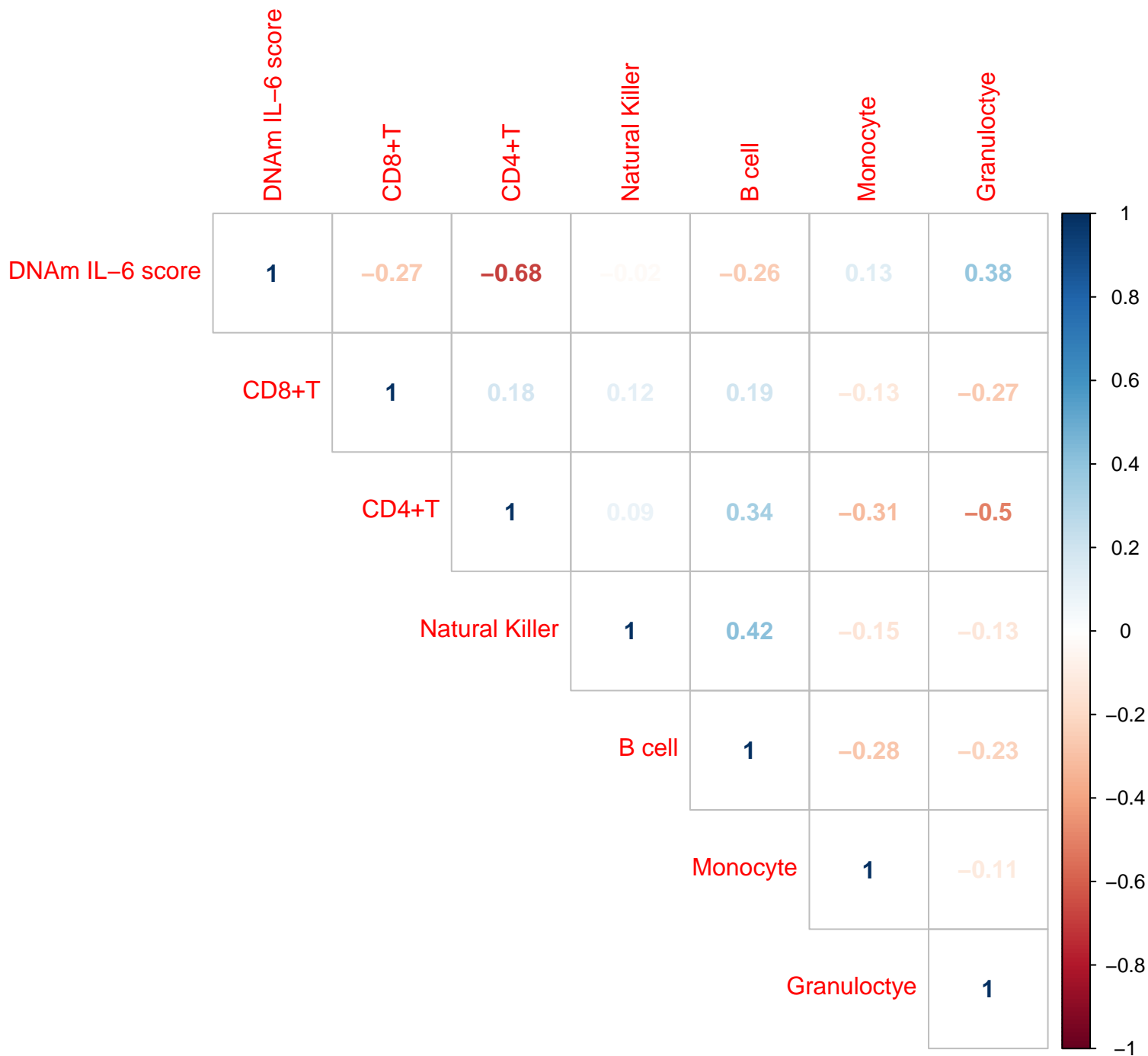

Supplement: glab046_suppl_Supplementary_eFigure2 [file glab046_suppl_supplementary_efigure2.pdf]
