# Supplementary material for: Creating and Validating a DNA Methylation-Based Proxy for Interleukin-6
Source: J Gerontol A Biol Sci Med Sci. 2021 Feb 17;76(12):2284–92. doi: 10.1093/gerona/glab046 (PMC8599002; doi:10.1093/gerona/glab046)

**Supplement.**

**eTable 1. Elastic net regression CpGs and corresponding weights for IL-6 in the Lothian Birth Cohort 1936**

**eTable 2. Association between Olink IL-6 and traits previously correlated with circulating IL-6 in the Lothian Birth Cohort 1936 (Wave 1; n=886)**

**eTable 3. Pearson correlations and variance explained by each individual CpG site comprising the DNAm IL-6 score in relation to serum IL-6 in Generation Scotland**

**eFigure 1. Pearson correlations between measured log(IL-6), the DNAm IL-6 score and the DNA methylation-based smoking score in Generation Scotland**

**eFigure 2. Pearson correlations between the DNAm IL-6 score and the imputed cell proportions in Generation Scotland**

**eTable 1.** **Elastic net regression CpGs and corresponding weights for IL-6 in the Lothian Birth Cohort 1936**

| ***CpG*** | ***Elastic net coefficient*** | ***Correlation with IL-6*** | ***Chromosome*** | ***Position*** | ***UCSC Gene Name*** | ***EWAS catalogue*** |
| --- | --- | --- | --- | --- | --- | --- |
| cg26144437 | 0.054 | 0.14 | chr1 | 145474469 | *ANKRD34A* | - |
| cg03076319 | 0.38 | 0.14 | chr2 | 238395764 | *MLPH* | HIV infection |
| cg05575921 | -0.14 | 0.16 | chr5 | 373378 | *AHRR* | smoking (p=1.4x10^-206^); maternal smoking in pregnancy |
| cg21368161 | 0.12 | 0.15 | chr7 | 1596136 | *TMEM184A* | Foetal vs. adult liver; gestational age |
| cg04642923 | 0.25 | 0.14 | chr11 | 33398366 |  | Clear cell renal carcinoma |
| cg21123519 | 0.0056 | 0.12 | chr14 | 69095679 |  | Foetal vs. adult liver |
| cg04583842 | 0.00022 | 0.16 | chr16 | 88103117 | *BANP* | Age 4 vs. age 0; smoking (p=3.6x10^-28^); gestational age |
| cg16357224 | 0.0036 | 0.13 | chr16 | 86530053 |  | HIV infection |
| cg24455236 | 0.064 | 0.13 | chr17 | 40824361 | *PLEKHH3* | Age 4vs. age 0; gestational age; sex |
| cg25323809 | 0.19 | 0.13 | chr19 | 34663190 | *LSM14A* | Maternal weight |
| cg03885055 | -0.45 | 0.17 | chr1 | 16723232 | *C1orf144* | Clear cell renal carcinoma; pancreatic ductal carcinoma; BMI; gestational age |
| cg17412005 | -0.088 | 0.18 | chr1 | 45806886 | *MUTYH;TOE1* | Primary Sjorgens syndrome; gestational age; smoking (p=1.7x10^-6^) |
| cg19638572 | 0.36 | 0.17 | chr1 | 206733139 | *RASSF5* | Foetal vs. adult liver; clear cell renal carcinoma; gestational age; high density lipoprotein |
| cg04921989 | 0.086 | 0.14 | chr2 | 132183100 |  | pancreatic ductal adenocarcinoma |
| cg14965639 | -0.11 | 0.17 | chr2 | 48795994 | *STON1-GTF2A1L* | gestational age; pancreatic ductal adenocarcinoma |
| cg04381957 | -0.019 | 0.16 | chr3 | 16550461 | *RFTN1* | Age 4 vs. age 0; foetal vs. adult liver; clear cell renal carcinoma; age; smoking (p=1.1x10^-6^) |
| cg20789595 | -0.31 | 0.16 | chr3 | 123063477 | *ADCY5* | Clear cell renal carcinoma |
| cg24935598 | 0.091 | 0.15 | chr3 | 193921489 |  | Gestational age |
| cg26230601 | -0.11 | 0.15 | chr3 | 194072216 | *CPN2* | Maternal BMI |
| cg16508480 | 0.029 | 0.13 | chr5 | 5139874 | *ADAMTS16* | Pancreatic ductal adenocarcinoma; HIV infection; age group |
| cg12503394 | 0.0011 | 0.15 | chr7 | 1596118 | *TMEM184A* | Fetal vs. adult liver; gestational age; smoking (p=5.9x10^-7^) |
| cg18925601 | -0.018 | 0.13 | chr7 | 158752715 |  | - |
| cg19584649 | 0.081 | 0.15 | chr7 | 158607898 | *ESYT2* | - |
| cg03245734 | 0.021 | 0.13 | chr8 | 108510096 | *ANGPT1* | Clear cell renal carcinoma; rheumatoid arthritis |
| cg04366687 | 0.082 | 0.13 | chr8 | 145107199 | *OPLAH* | Fetal vs. adult liver |
| cg14044707 | 0.0075 | 0.13 | chr9 | 132628998 | *USP20* | Gestational age; smoking (p=2.3x10^-5^) |
| cg25250132 | 0.24 | 0.16 | chr10 | 43704423 | *RASGEF1A* | smoking (p=1.4x10^-9^) |
| cg04468741 | 0.025 | 0.13 | chr11 | 12181467 | *MICAL2* | Pancreatic ductal adenocarcinoma; gestational age |
| cg23729763 | 0.10 | 0.16 | chr11 | 129991544 | *APLP2* | Age group; gestational age |
| cg10195814 | 0.063 | 0.13 | chr12 | 10560602 | *KLRC4* | - |
| cg03998636 | -0.051 | 0.15 | chr13 | 111210121 | *RAB20* | Gestational age |
| cg00768409 | 0.11 | 0.13 | chr15 | 83954392 | *BNC1* | Sex |
| cg20059928 | -0.16 | 0.15 | chr15 | 40361485 |  | Age 4 vs. age 0; smoking (1.1x10^-76^); fetal vs. adult liver; gestational age |
| cg04928129 | -0.15 | 0.16 | chr16 | 1429051 | *UNKL* | HIV infection |
| cg12929678 | 0.46 | 0.18 | chr18 | 67624393 | *CD226* | Gestational age |

**eTable 2. Pearson correlations and variance explained by each individual CpG site comprising the DNAm IL-6 score in relation to serum IL-6 in Generation Scotland**

| **CpG** | **Pearson correlation** | **P** | **Variance explained (%)** |
| --- | --- | --- | --- |
| cg17412005 | 0.23 | 2.4x10^-6^ | 5.23 |
| cg04583842 | 0.22 | 6.3x10^-6^ | 4.80 |
| cg04381957 | 0.22 | 9.1x10^-6^ | 4.64 |
| cg21368161 | 0.21 | 1.2x10^-5^ | 4.53 |
| cg14965639 | 0.17 | 0.00042 | 2.96 |
| cg19638572 | 0.17 | 0.0005 | 2.89 |
| cg05575921 | 0.16 | 8.4x10^-4^ | 2.65 |
| cg12503394 | 0.16 | 0.0015 | 2.41 |
| cg04366687 | -0.15 | 0.0023 | 2.21 |
| cg04928129 | 0.12 | 0.014 | 1.44 |
| cg03076319 | 0.11 | 0.023 | 1.23 |
| cg23729763 | 0.10 | 0.033 | 1.09 |
| cg10195814 | 0.097 | 0.047 | 0.94 |
| cg12929678 | 0.094 | 0.056 | 0.87 |
| cg20789595 | 0.091 | 0.063 | 0.83 |
| cg03245734 | -0.089 | 0.069 | 0.79 |
| cg03998636 | 0.087 | 0.076 | 0.76 |
| cg24935598 | -0.078 | 0.11 | 0.61 |
| cg25250132 | 0.077 | 0.12 | 0.59 |
| cg24455236 | 0.051 | 0.30 | 0.26 |
| cg04921989 | -0.046 | 0.35 | 0.21 |
| cg19584649 | 0.045 | 0.36 | 0.21 |
| cg26230601 | -0.043 | 0.38 | 0.19 |
| cg14044707 | 0.044 | 0.37 | 0.19 |
| cg25323809 | -0.038 | 0.44 | 0.14 |
| cg26144437 | 0.030 | 0.54 | 0.092 |
| cg00768409 | -0.029 | 0.55 | 0.087 |
| cg18925601 | 0.029 | 0.55 | 0.087 |
| cg21123519 | -0.024 | 0.62 | 0.059 |
| cg04468741 | 0.021 | 0.66 | 0.046 |
| cg20059928 | 0.017 | 0.73 | 0.028 |
| cg04642923 | 0.012 | 0.80 | 0.015 |
| cg16508480 | -0.0086 | 0.86 | 0.0075 |
| cg03885055 | -0.0039 | 0.94 | 0.0015 |
| cg16357224 | 0.0037 | 0.94 | 0.0014 |

**eTable 3. Association between Olink IL-6 and traits previously correlated with circulating IL-6 in the Lothian Birth Cohort 1936 (Wave 1; n=886)**

Log odds are presented for smoking. Significant associations are highlighted in bold. BMI=body mass index; SIMD=Scottish Index of Multiple Deprivation.

|  | ***β*** | ***SE*** | ***P*** |
| --- | --- | --- | --- |
| BMI | 0.14 | 0.03 | 3.5x10^-5^ |
| SIMD | -0.094 | 0.03 | 0.0043 |
| Alcohol | -0.059 | 0.03 | 0.062 |
| Smoking | 0.21 | 0.07 | 0.00099 |

**eFigure 1. Pearson correlations between measured log(IL-6), the DNAm IL-6 score and the DNA methylation-based smoking score in Generation Scotland.**


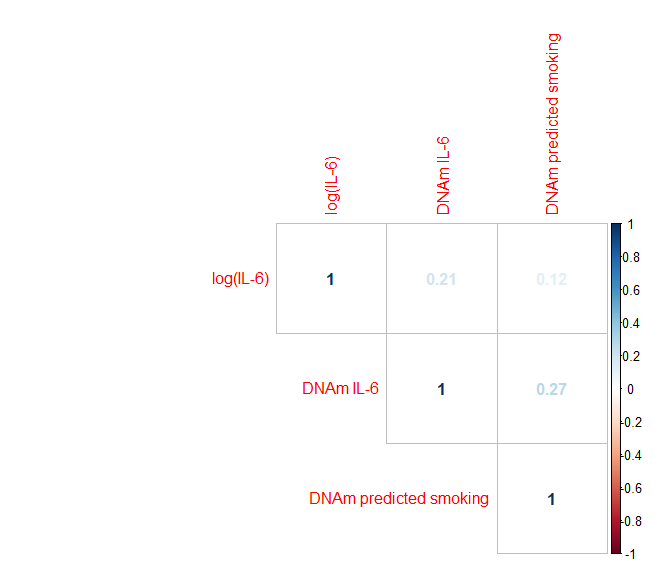


**eFigure 2. Pearson correlations between the DNAm IL-6 score and the imputed cell proportions in Generation Scotland.**


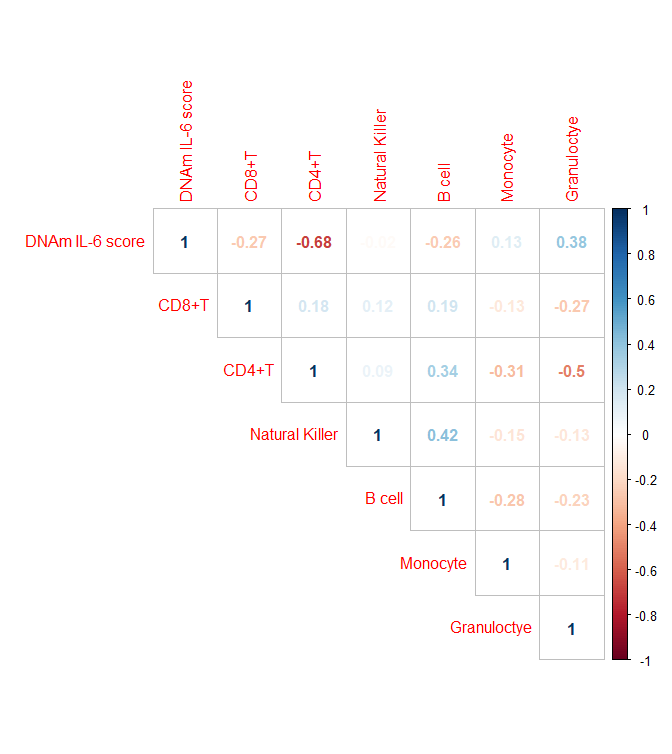

Supplement: glab046_suppl_Supplementary_Material [file glab046_suppl_supplementary_material.docx]
